# Biological Aging Modulates Cell Migration via Lamin A/C-Dependent Nuclear Motion

**DOI:** 10.3390/mi11090801

**Published:** 2020-08-24

**Authors:** Jung-Won Park, Seong-Beom Han, Jungwon Hah, Geonhui Lee, Jeong-Ki Kim, Soo Hyun Kim, Dong-Hwee Kim

**Affiliations:** 1 KU-KIST Graduate School of Converging Science and Technology, Korea University, Seoul 02841, Korea; jungwonpark@korea.ac.kr (J.-W.P.); himne10@korea.ac.kr (S.-B.H.); jwhah@korea.ac.kr (J.H.); sharpafm@korea.ac.kr (G.L.); kjk9203@korea.ac.kr (J.-K.K.); soohkim@kist.re.kr (S.H.K.); 2 Center for Biomaterials, Biomedical Research Institute, Korea Institute of Science and Technology, Seoul 02792, Korea

**Keywords:** aging, cell motility, lamin A/C, nuclear dynamics

## Abstract

Aging is a progressive functional decline in organs and tissues over time and typically represents the accumulation of psychological and social changes in a human being. Diverse diseases, such as cardiovascular, musculoskeletal, and neurodegenerative disorders, are now understood to be caused by aging. While biological assessment of aging mainly focuses on the gradual changes that occur either on the molecular scale, for example, alteration of gene expression and epigenetic modification, or on larger scales, for example, changes in muscle strength and cardiac function, the mechanics that regulates the behavior of individual cells and interactions between the internal elements of cells, are largely missing. In this study, we show that the dynamic features of migrating cells across different human ages could help to establish the underlying mechanism of biological age-dependent cellular functional decline. To determine the relationship between cellular dynamics and human age, we identify the characteristic relationship between cell migration and nuclear motion which is tightly regulated by nucleus-bound cytoskeletal organization. This analysis demonstrates that actomyosin contractility-dependent nuclear motion plays a key role in cell migration. We anticipate this study to provide noble biophysical insights on biological aging in order to precisely diagnose age-related chronic diseases.

## 1. Introduction 

Aging is a complex and multifaceted time-dependent biological process associated with the decline of cellular functions, onset of diverse diseases, and incremental risk of death [1]. Accumulation of organ-specific aging processes causes progression of cardiovascular, musculoskeletal, and neurodegenerative disorders, as well as, various chronic diseases, such as diabetes, hypertension, and Alzheimer’s disease [2]. According to previous studies on the pathological aspects of these diseases, they commonly involve dysfunction of cellular or subcellular organelles, for example, telomere shortening, mitochondrial dysfunction, cell senescence, stem cell depletion, and genetic alterations [3].

Prevention of age-related diseases, therefore, requires an in-depth understanding of the underlying cellular mechanisms of aging. Previous studies involving aging have mainly focused on phenotypic alterations at the organ and tissue level or dysfunction of multicellular organs, for example, reduction of mechanical strength, barrier function, and energy production in aged skin [4], and a dramatic decline of cardiovascular function in aged heart [5]. Epigenetic alterations via DNA methylation also vary with age [6]. However, the mechanism of how biological aging determines the dynamic motion of individual cells that play a critical role in organ-specific functionality is not yet clearly identified. 

Cell migration is one of the most systematically characterized dynamic features of cellular function and it is universally involved in tissue renewal, immune responses, and pathological progression of diseases [7,8,9]. In particular, mesenchymal cell migration consisting of stepwise processes, i.e., protrusion of the leading edge, adhesion formation, and contraction of the cell body followed by detachment of the rear end, has been extensively studied [10], with the finding that cytoskeletal organization is the critical subcellular organization that mediates the individual steps of cell migratory machinery [11]. For instance, establishment of cell polarity largely depends on reorganization of microtubules [12], intermediate filaments modulate focal adhesion dynamics [13], and actin filaments engage in protrusion, adhesion, contraction, and retraction with assistance from myosin motors [14].

While a series of steps constructing the whole cell motion exquisitely represent the mechanical interplay between cellular machinery and the extracellular microenvironment, the most recent discoveries have shown that cytoskeletal architectures are not isolated from the cell nucleus, but they are instead closely involved in biophysical signal pathways traversing between the extracellular microenvironment and the intranuclear space through linkers of the nucleoskeleton to the cytoskeleton (LINC) molecular complexes [15,16]. 

LINC-mediated mechanotransduction utilizes SUN-KASH molecular interactions, which are essential to connect nuclear lamina and extranuclear cytoskeletal organizations [17]. The nuclear lamina, a component of the nuclear envelope, is composed of two types of lamin proteins, namely, A-type for lamin A/C and B-type for lamin B1 and B2. In contrast to B-type lamin proteins that mediate structural integrity of the nucleus via elastic behavior [18], A-type lamin proteins directly interact with intranuclear chromosomal organization by altering chromatin dynamics allowing them to regulate gene expression [19,20,21]. 

The underlying molecular connection between the nucleus and the cytoskeleton suggests that the nucleus is another important subcellular organelle that regulates cell migration [22]. For instance, nuclear re-positioning inside a motile cell is essential during polarization to initiate cell migration [23], and nuclear shape change is highly synchronized with distinct cell migration modes that compose the whole cell migratory cycle [24]. Accordingly, monitoring of the nuclear shape enables prediction of migratory potential, as well as the direction of a moving cell [24]. 

In this study, therefore, we examine how biological aging alters the cell–nucleus interaction that ultimately differentiates cell migration. Moreover, we aim to identify the underlying mechanism regarding how age-associated disruption of nuclear structures switches cell migration.

## 2. Materials and Methods

### 2.1. Cell Culture

Human dermal fibroblast (HDF) cell lines from 2-(GM00969), 3-(GM05565), 85-(AG09558), and 92-(AG09602) year-old donors (Coriell Institute for Medical Research, Camden, NJ, USA) were cultured in Dulbecco’s modified Eagle’s medium (DMEM, Corning, Manassas, VA, USA) supplemented with 10% fetal bovine serum (FBS, Gibco, Grand Island, NY, USA) and 1% penicillin/streptomycin solution (Gibco, Grand Island, NY, USA) maintained at 37 °C in a humidified, 5% CO_2_ environment. HDFs were seeded at a density of 1 × 10^5^ cells/mL (~30 cells/mm^2^) on a glass-bottom dish (MatTek, Ashland, MA, USA) and cultured for 12 h to analyze cell characteristics. The glass bottom dish was coated with 200 μL of collagen solution (3.18 µg/cm^2^), including 13.8 μL of type-I rat-tail collagen (Corning, Bedford, MA, USA) diluted in 1 mL of 0.2 N acetic acid for 8 h, at 4 °C before cells were seeded.

### 2.2. Immunofluorescence Microscopy

HDFs were fixed with 4% paraformaldehyde (Electron Microscopy Sciences) at room temperature, for 10 min. Then, tritonX-100 was treated for permeabilization of the fixed cells at room temperature, for 10 min. After washing with phosphate-buffered saline (PBS) containing 0.1% bovine serum albumin (BSA), cells were incubated with 3% BSA at room temperature for 1 h to block nonspecific binding. For lamin A/C staining, cells were treated with anti-lamin A/C antibody (1:200, EMD Millipore) for 1 h, at room temperature, then incubated with Alexa Fluor 594 goat-anti mouse (1:500, benthyl), for 1 h at room temperature. DAPI (300 nM, Invitrogen) and Alexa-Fluor phalloidin (1:200, Invitrogen) were treated to stain nuclear DNA or F-actin organization, respectively. Then, the cells were washed with PBS 3 times and immunofluorescence image were obtaining using a Nikon A1R confocal microscope through a Plan Apo VC 60× Oil lens or plan 20× lens.

### 2.3. Morphometric and Motility Analysis 

We used Metamorph software (Molecular Devices, San Jose, CA, USA) for the morphometric analysis of the cells and nuclei (i.e., size and shape factor). To analyze cell motility (i.e., average and persistence speed) in HDFs from young and old donors, x and y coordinates and time intervals were obtained by tracking single HDFs every 2 min, for 8 h. Then, we calculated two types of speed factor using average and persistence vectors. Average instantaneous speed was calculated by dividing total traveled distance by cell tracking time. Persistence vectors and end-to-end distance showed how cells maintained persistent motion defined as the cell traveling length (>10 µm) in the moving direction within 70° [25]. At least 20 cells were analyzed per each condition. To assess nuclear movement, nuclei and nucleoli were traced in each time frame (4 min) to obtain the centroid positions. Nuclear translocation was calculated by averaging the displacement of the nuclear centroids, while the nuclear rotational angle was determined by the inner product of centroid-pointing vectors between the nucleus and nucleolus.

### 2.4. Laser Ablation and Actin Retraction Measurement

To investigate actomyosin contractility in HDFs derived from young and old donors, we used a Nikon A1R confocal microscope with a Plan Apo VC 60× Oil lens for every second. To visualize GFP-actin fiber, we scanned the sample with the 488 nm laser at a scan speed of 0.5 frame/s and a scan size of 1024 pixel. Laser ablation of actin stress fiber was conducted by stimulation of 406 nm laser with 30 high voltage (HV) power at a scan speed of 32 frame/s for 10 s. To perform the experiment, cells were maintained in a 5% CO_2_ atmosphere at 37 °C using a microscope stage incubator (Okolab, Naples, Italy). Retraction of actin stress fiber was measured using NIS-elements software (Nikon). More than 15 actin stress fibers were measured to assess retraction distance.

### 2.5. Quantitative Image Analysis 

The molecular content of lamin A/C in a single cell resolution was assessed by high-throughput cell phenotyping [26], and immunofluorescence images were captured with a Cytation 3 imaging reader (BioTek). Each young and old HDF stained with DAPI and anti-lamin A/C antibody was autofocused on the DAPI-stained nucleus; fluorescence images were automatically taken in a 10 × 10 montage in two channels using DAPI and GFP filter cubes through a 10× plan lens. To identify the nuclear region in the acquired images, DAPI channel images were used to segment each nucleus by applying the same threshold to all images. To quantify the lamin A/C content at the single cell level, fluorescence intensity levels in the raw images corresponding to the lamin A/C channel were calculated based on the segmented nuclear regions following the above procedure. All image processes were carried out using a customized MATLAB code.

### 2.6. Data Processing and Statistical Analysis 

A two-tailed unpaired Student’s t-test was conducted using Graphpad Prism (Graphpad Software, Version 5.03, San Diego, CA, USA). Error bars represent the standard error of the mean (S.E.M.) of averaged values, unless mentioned otherwise. After confirming a normal distribution of the collected dataset, statistical significance was further assessed using the unpaired t-test. ***, *p* < 0.0001; **, *p*< 0.01, *, *p* < 0.05, and NS, no significance (*p* > 0.05).

## 3. Results and Discussion

### 3.1. Biological Aging Declines Cell Migration

**Figure 1:**
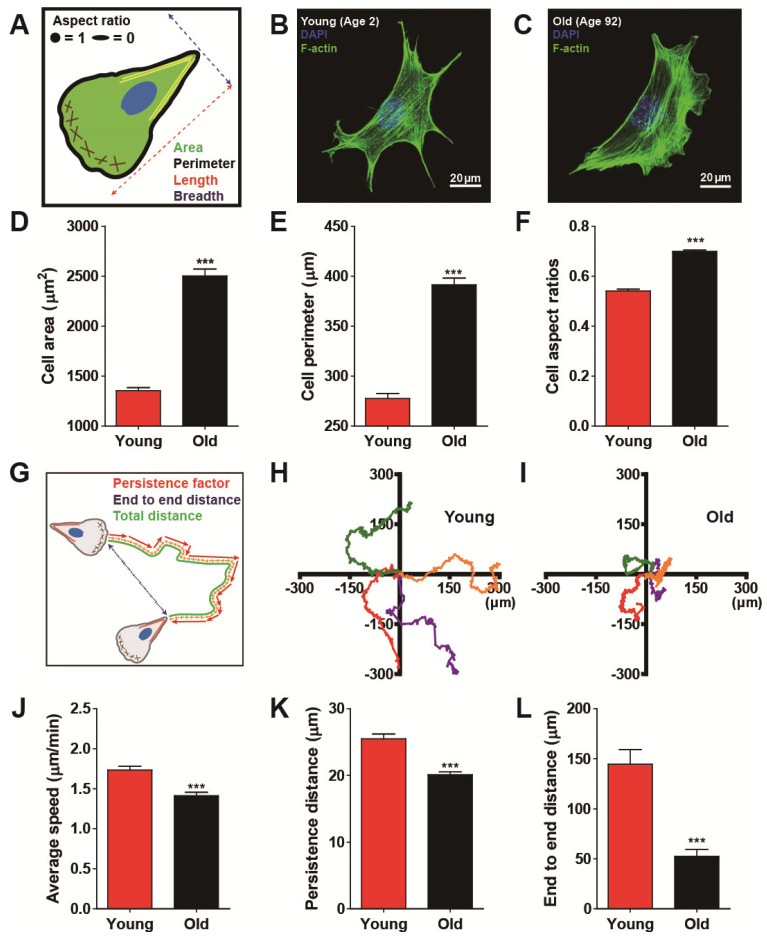
Biological age-dependent changes of cell morphology and motility. (**A**–**F**) Age-dependent distinct cell morphology. Schematic defining of single cell morphology (**A**). Representative immunofluorescence confocal microscopy of young (age < 5) and old (age > 80) human dermal fibroblasts (HDFs) displaying F-actin (green) and nucleus (DAPI, blue) (**B**,**C**). Note that young and old cells depict HDFs obtained from 2- to 3- year-old cohorts and 85- to 92- year- old cohorts, respectively. Old cells are larger (**D**,**E**) and less elongated (**F**) than young cells. Cell aspect ratio is defined as W/L, where L is the longest chord of the cell and W is the caliper width, perpendicular to the length, approaching 1 for a less elongated and relatively symmetric cell shape and 0 for an elongated cell shape. More than 600 cells were analyzed for each condition. (**G**–**L**) Age-dependent alteration of cell motility. Schematic definition of single cell motility illustrates cell trajectory (orange), persistence vectors (red), and end-to-end distance (blue) of HDFs migrating on a type-I rat-tail collagen coated glass coverslips (**G**). Representative cell trajectories of young (**H**) and old HDFs (**I**) displayed distinct cell migration depending on cellular age. Systematic cell tracking based on time-lapse cell monitoring reveal that biological aging significantly reduces cell migrating speed (**J**) and migratory persistence (**K**,**L**). Note that average speed is the total distance traveled by the cell for 8 h of total monitoring time (**J**), indicating instantaneous motility of the cell, while persistence distance assessed by an average length of persistence vectors (**K**) and end-to-end distance (**L**) implies the persistence of cell migration. More than 50 cells were analyzed for each condition. In panels (**D**–**F**) and (**J**–**L**), error bars represent S.E.M. of average values and statistical significance was assessed by the unpaired *t*-test; *** *p* < 0.001.

Biological aging commonly involves functional decline in organs that are hierarchically developed and organized from multiple cells. Since defects in cellular biophysical properties are hallmarks of malfunctioning cells, we first tested whether cells apparently change their morphology in response to biological aging. We conducted morphometric analysis on human dermal fibroblasts (HDFs) obtained from healthy donors ranging in age from 2 to 92 years (see more details on cell culture in Materials and Methods). Since fibroblasts adherent to collagen-coated glass substrates generally form well-organized actin stress fibers on the basal surface of the cell [27], descriptors of cell morphology, such as area and perimeter, were collected from F-actin-stained images (Figure 1A–C and Supplemental Figure S1). Systematic comparison of cell morphology clearly depicted that old cells (i.e., HDFs from donors aged >80 years) were larger and more symmetrical in shape than young cells (i.e., HDFs from donors aged <5 years) (Figure 1D–F and Supplemental Figure S1).

Since we previously showed that migrating cells dynamically change their morphology [24], which is significantly altered with respect to age (Figure 1A–F and Supplemental Figure S1), we hypothesized that differently aged cells could display distinct cell migration. To this notion, we tracked randomly migrating cells, which revealed significantly different migrating patterns with respect to age, where young cells were not only motile but strongly maintained their motility as compared with old cells (Figure 1G–I). Quantification of cell motility by comparing total traveled distance in a given time (i.e., average speed) confirmed the notion that young cells moved significantly faster than old cells (Figure 1J and Supplemental Figure S2A). Moreover, to assess cellular ability to maintain motility, we analyzed cell persistence, where persistence distance was estimated by the average length of persistence vectors (see more details on persistence vectors in Materials and Methods). As expected, young cells moved more persistently, i.e., they tended to maintain their migrating patterns more strongly than old cells (Figure 1K and Supplemental Figure S2B), thereby, traveling much further in a given time (Figure 1L and Supplemental Figure S2C). These results suggest that biological aging reduces random cell migration. 

### 3.2. Age-Dependent Nuclear Morphology Distinguishes Cell Migration

**Figure 2:**
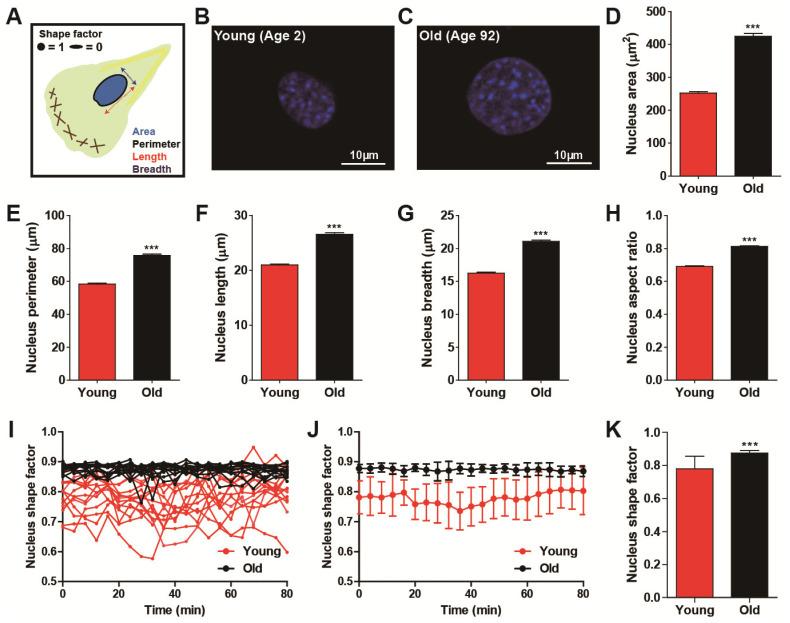
Age-dependent alteration of nuclear morphology. (**A**–**H**) Morphometric comparison of nuclear morphology between young (age < 5) and old (age > 80) HDFs. Schematic defining of descriptors of nuclear morphology (**A**). Note that shape factor is defined as 4π(area)/(perimeter)^2^, and thus, the nucleus shape factor of elongated and round nuclei approaches 0 and 1, respectively. Representative immunofluorescent images of nuclei of young and old HDFs stained with DAPI (**B**,**C**). Nuclei of young cells are smaller (**D**–**G**) and more elongated (**H**) than nuclei of old cells. More than 600 cells were analyzed for each condition. (**I**–**K**) Time-lapse monitoring of nuclear shape. Fluctuation of nuclear shape changes in young cells is larger than changes in nuclear shape in old cells (**I**,**J**). Nuclei were imaged using time-lapse fluorescence microscopy, every 4 min, for 80 min. Time-lapse monitoring of nucleus shape factor (**I**) and fluctuation of their average values (**J**) are presented. Average values of time-lapse monitored nuclear shape factor indicate nuclei of young cells are more elongated and they change their shape more dynamically than nuclei of old cells (**K**). More than 10 cells were monitored for each condition (young, 13 cells and old, 15 cells). In panels (**D**–**H**), error bars indicate S.E.M. and statistical differences were calculated using the unpaired t-test; *** p < 0.001. In panels (**J**) and (**K**), error bars indicate standard deviation (S.D.) and statistical differences were calculated using the unpaired *t*-test. *** p < 0.001.

As shown previously, migrating fibroblasts dynamically alter their shape and nuclear morphology with high fidelity [24], and cells obtained from differently aged donors showed distinct cell migration (Figure 1). Therefore, we examined whether nuclear morphology could be a biophysical descriptor of cellular age in a randomly migrating cell. To systematically compare nuclear size and shape, we characterized nuclear surface area, perimeter, length, breadth, and aspect ratio by applying a high-throughput cell phenotyping algorithm on DAPI-stained nuclei (Figure 2A–C). Consistent with results from cell morphometry (Figure 1A–F), old cells typically displayed large nuclear size as compared with the nuclei of young cells (Figure 2D–G and Supplemental Figure S3), and they were rounder and more symmetrical in shape (Figure 2H). 

Since alteration of nuclear morphology during cell migration may distinguish cell migratory modes [24], we further monitored alterations in nucleus shape, which was a more accurate way to characterize dynamically switching nuclear morphology between elongated and round shape (Figure 2I–K) [28]. Time-lapse live cell tracking further revealed that the nuclei of young HDFs experienced high fluctuations in nucleus shape factor between elongated morphology (i.e., shape factor approaching 0) and round morphology (i.e., shape factor approaching one), while the nuclei of old HDFs maintained their round nuclear shape without large variation (Figure 2I,J). Consequently, the averaged nucleus shape factors showed not only a significant reduction but a large error bar in the nuclei of young donors (Figure 2K), demonstrating dynamic alteration in the nuclear morphology of young cells as compared with the nuclear shape of old cells (Figure 2J,K).

Consistent with our previously reported multiparametric functional relationship between nuclear morphology and cell migration, where fast-migrating cells displayed an elongated nuclear shape [24], these results further indicate that age-dependent nuclear morphology is highly synchronized with cell migration, where the nuclei of young cells follow the phenotypic features of fast-migrating cell nuclei, whereas while nuclei of old cells tend to exhibit the nuclear morphology of slowly migrating cells.

### 3.3. Distinct Nuclear Motility Synchronizes with Biological Ages 

**Figure 3:**
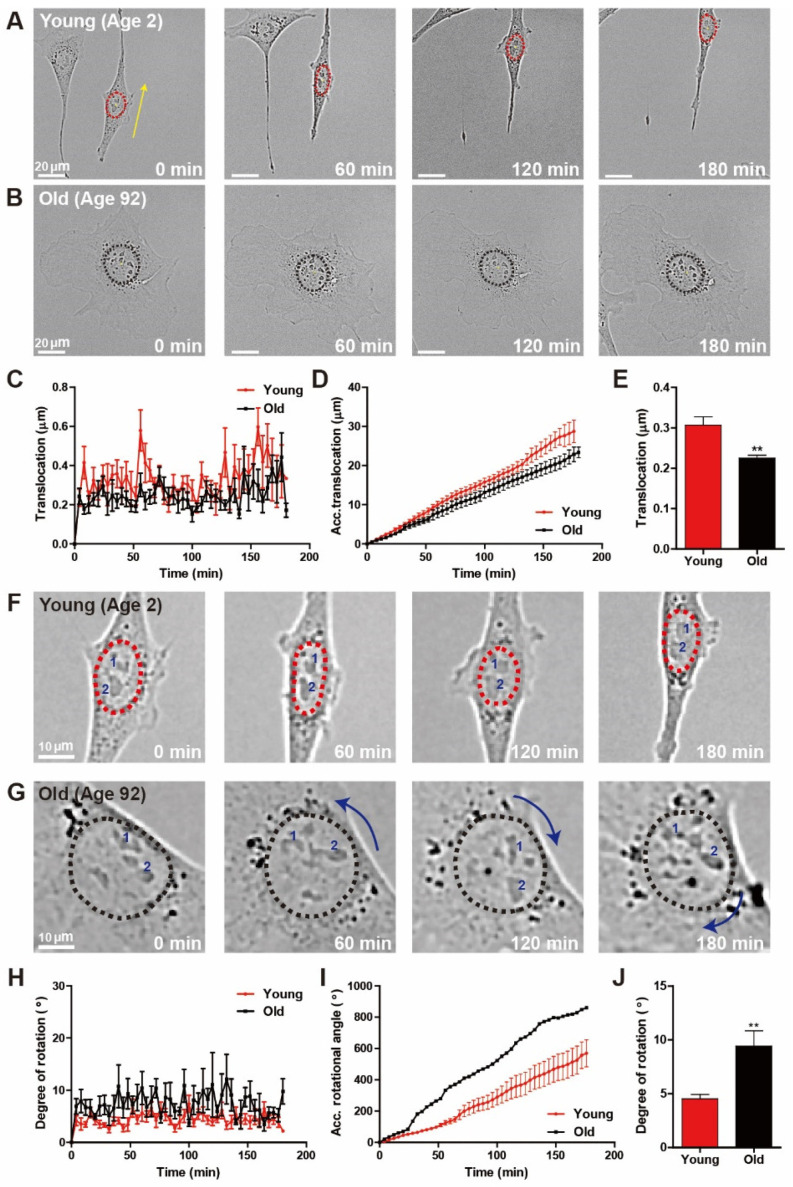
Age-dependent distinct nuclear motion. (**A**–**E**) Time-lapse monitoring of nuclear translocation during random migration of young and old HDFs. Elongated nuclei of young HDFs showed translocation, while nuclear rotation was dominant in round nuclei of old HDFs (**A**,**B**). Dotted lines delineate the nuclear boundary and arrows represent the magnitude of longitudinal displacement of nuclei. Quantification of displacement of nuclear centroids every 4 min for 3 h indicated that nuclei of young HDFs underwent more significant translocation than nuclei of old HDFs (**C**–**E**). (**F**–**J**) Time-lapse monitoring of nuclear rotation during random migration of young and old HDFs. Nuclear rotation was more frequently observed in old HDFs displaying round nuclear shape than that of nuclei of young HDFs typically displaying elongated nuclear shape (**F**,**G**). Dotted lines delineate nuclear boundaries, numbers inside the nuclei indicate the nucleoli, and curved arrows represent the magnitude of the rotational angle of the nuclei. The rotational angle quantified by the inner product of the centroid-pointing vectors of the nuclei and nucleoli indicate that nuclear rotation was more dominant in old HDFs (**H**–**J**). See more details on quantification of nuclear rotation in Materials and Methods. In panels (**C**–**E**) and (**H**–**J**), >10 cells were analyzed for each condition. Error bars indicate S.E.M. and statistical differences were calculated using the unpaired *t*-test. ** p < 0.01.

Migratory fibroblasts placed on a flat surface systematically switch between directional migration mode and a less motile hesitation mode, typically displaying elongated nuclear shape and round nuclear shape, respectively [24]. Since nuclear shape is also highly intertwined with distinct nuclear motion during cell migration, we analyzed whether age-dependent alterations in cell motility could differentiate nuclear motion. First, we characterized nuclear translocation in migrating HDFs of young and old age, where time-lapse live-cell tracking was applied to measure the centroid displacement of the nucleus during random cell migration (Figure 3). While nuclei of young HDFs revealed the directional, persistent translocation along the direction of cell migration, nuclear translocation of old HDFs barely occurred (Figure 3A,B). Quantification of distances that the nucleus traveled further confirmed rapid displacement of young HDF nuclei (Figure 3C–E).

Since nuclear rotation is the other distinct nuclear motion typically observed when randomly migrating cells feature less motile phenotype, next, we compared the rotational angles of the nuclei of young and old HDFs. In contrast to nuclear translocation, nuclear rotation is more dominant in old HDFs, as young HDF nuclei rarely showed rotational motion (Figure 3F,G). To quantitatively compare nuclear rotation between young and old cells, we calculated the angle of nuclear rotation from the inner products of centroid-pointing vectors of nuclei and individual nucleoli, confirming that the degree of nuclear rotation (i.e., nuclear rotational angle) in old cells was larger than that in young cells (Figure 3H). Consequently, the accumulative nuclear rotation angle in old cells increased more rapidly than in young cells (Figure 3I), and the average degree of nuclear rotation was significantly larger in old cells (Figure 3J).

Together, these results suggest that differently aged cells exhibit distinct nuclear motion. Furthermore, biological aging could induce the transition of nuclear motion during random cell migration, where nuclear translocation is dominant in fast migrating young cells and nuclear rotation is dominant in slowly migrating old cells.

### 3.4. Nulcear Lamin A/C Mediated Actomyosin Contractility Determines Age-Dependent Cell Migration

**Figure 4:**
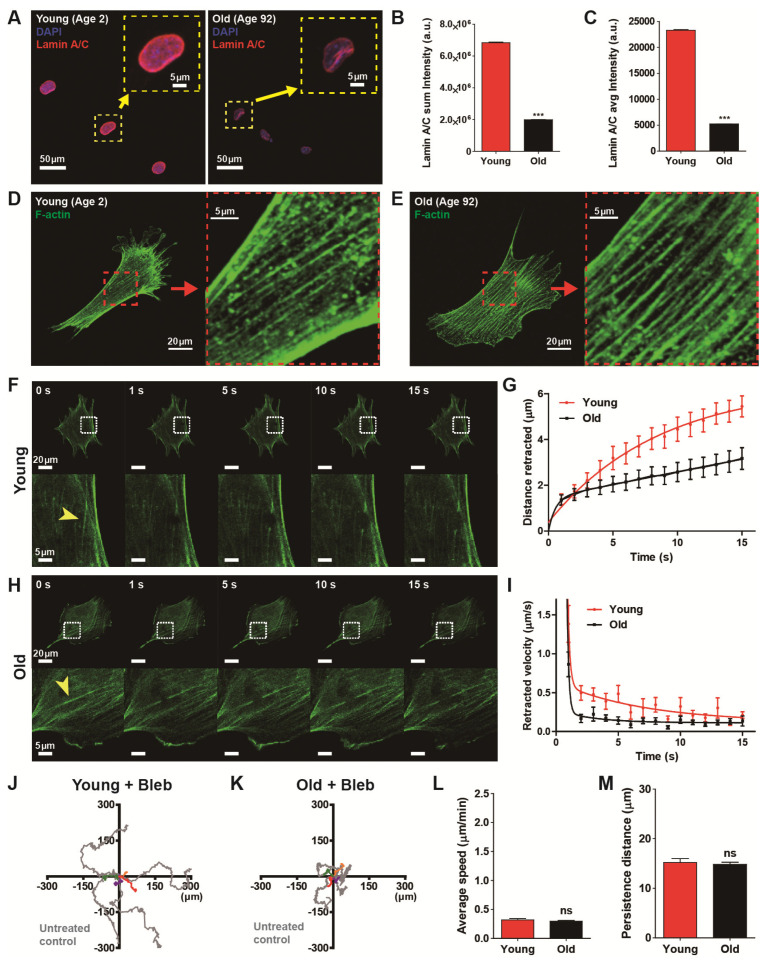
Actomyosin contractility determines age-dependent cell migration. (**A**–**C**) Differential molecular contents of lamin A/C in young and old HDFs. High-throughput immunofluorescence analysis indicated enhanced molecular content of lamin A/C in the nuclei of young HDFs as compared with the nuclei of old HDFs (**A**). Insets show representative lamin A/C staining (red) along the nuclear boundary in HDFs. Statistical quantification revealed that lamin A/C expression in old HDFs decreased fourfold as compared with young HDFs (**B**,**C**). More than 2900 cells (young, 2956 cells and old, 4433 cells) were analyzed for each condition. (**D**–**I**) Biophysical measurement of actomyosin contractility by laser ablation-induced monitoring of F-actin retraction. Organization of actin stress fibers in young and old HDFs did not show any structural differences (**D** vs. **E**). Monitoring of F-actin retraction after laser ablation of actin stress fibers in young HDFs and old HDFs (**F**–**I**). Arrowheads indicate the position of laser irradiation (**F**,**H**), where actin stress fibers were bifurcated immediately after laser stimulation and progressively retracted to opposite sides. Time-lapse monitoring displayed retraction distance and retraction speed in young HDFs (*n* = 6) were greater than in old HDFs (*n* = 11) (**G**,**I**). (**J**–**M**) Alteration of cell motility by inhibition of myosin activity. Cell motility was reduced by treating cells with blebbistatin, a myosin II ATPase-inhibitor, in young and old HDFs. Note that time-lapse monitoring of cell migration was performed for 8 h, 1 h after addition of 2 μM blebbistatin. Representative cell trajectories of young and old HDFs indicated cell migration was reduced by a decrease in myosin activity (**J**,**K**). Inhibition of myosin activity impedes cell migrationas compared with the results of Figure 1 by decreasing cell migration rate (**L**) and migratory persistence (**M**), regardless of aging. More than 20 cells were analyzed for each condition. In panels (**L**) and (**M**), error bars represent S.E.M. of averaged values and statistical significance was assessed by the unpaired *t*-test. *** p < 0.001; ns, no significance (*p* > 0.05).

To confirm the previous result showing age-dependent progressive decline of lamin A/C [29], we first estimated the molecular contents of lamin A/C caused by biological aging (Figure 4A–C). High-throughput cell phenotyping based on quantitative analysis of immunofluorescence revealed that expression of nuclear lamin A/C was significantly diminished in old HDFs as compared with young HDFs (Figure 4B,C). 

Cellular aging induced a decline of lamin A/C content (Figure 4A–C), which was closely related to the disruption of nucleus-bound F-actin organization, for example, perinuclear actin stress fibers [30,31] that tightly regulated cell migration [24]. Thus, we tested whether reduced expression of lamin A/C induced by natural aging also altered the F-actin architecture (Figure 4D,E). High-resolution confocal microscopy of F-actin stress fibers, however, did not show any notable structural remodeling (insets, Figure 4D,E), i.e., well-organized actin stress fibers were observed in both young and old HDFs. These results suggest that the organization of actin stress fibers are not the determinant of cell migration but the functionality of actin stress fibers could be more important in the modulation of age-dependent cell motility. 

Therefore, we hypothesized that lamin A/C content-dependent contractility of actin stress fibers could differ with biological aging. To this end, we assessed the cytoskeletal tension of actin stress fibers of young and old HDFs using laser ablation (Figure 4F–I). As described previously [32], an endogenous actin stress fiber was visualized in each green fluorescence protein (GFP)-actin transfected cell. When the laser was focused on a small area (~500 nm) in the middle of a randomly selected single F-actin fiber lying on the basal surface of the cell cultured on a collagen-coated glass bottom dish, the fiber was immediately (<1 s) ablated and progressively retracted to the opposite direction (Figure 4F,H). The distance of retraction of the actin stress fiber was 1.7-times larger in young HDF (5.44 μm) than in old HDF (3.17 μm) after 15 s of laser ablation (Figure 4G), i.e., actin cytoskeleton retraction was faster in young HDFs. These results indicate that cytoskeletal tension in young HDFs is higher than in old HDFs, and actomyosin contractility becomes weaker as the expression of lamin A/C diminishes with the aging process.

Therefore, we demonstrated that cell motility is significantly reduced by biological aging (Figure 1) and mechanical tension applied to actin stress fibers differs with the biological aging (Figure 4F–I). Since cell mobility depends on the contractile force generated by F-actin and myosin motors, we asked if biological age-dependent alteration of cell migration depends on actomyosin contractility. Therefore we monitored the trajectories of randomly migrating young and old cells after treatment of myosin-inhibiting blebbistatin to disrupt myosin activity (Figure 4J,K). Interestingly, we noted that inhibition of actomyosin contractility activity greatly reduced cell migration regardless of age (Figure 4L,M). Measurements of cell migration by average speed showed that motility of blebbistatin-treated cells was reduced in both young and old cells (Figure 4L). Both groups of cell migrated for a reduced distance in a given time compared to drug-free conditions and they showed no difference in migration speed between young and old cells, thereby, eliminating cellular motility dependence on biological age. Furthermore, the persistence of the cell migration, specifically observed in young cells, was also reduced by the myosin inhibitor, becoming similar in behaviors to old cells (Figure 4M). 

Consequently, these results decipher that underlying biophysical mechanism of lamin A/C-dependent reduced cell migration of aged cells is determined by myosin-dependent cytoskeletal tension.

## 4. Conclusions

Here, we show that the mechanics that regulate the behaviors of individual cells could explain pathological symptoms of biological aging that feature progressive attenuation of cellular function. Our results further indicate that cellular phenotypes across different human ages could reveal the underlying mechanism of age-induced cellular functional decline. These studies demonstrate that cell and nuclear dimensions that are tightly connected to modification of cell motility are altered in a highly predictive manner as biological aging progresses. Our results show that the dynamic feature of cell movement can establish a fundamental mechanism for the functional degradation of cells that depend on biological aging. To determine the relationship between cell dynamics and biological aging, we identified cell migration and the characteristic relationship between nuclear movements (i.e., translocation and rotation) that are closely related to aging. These results show that cell migration depends on nuclear motion and cell movement changes as biological aging progresses in a predictable way. By manipulating actomyosin contraction, we confirm and further identify the underlying mechanics of how cell migration is changed by an age-related decline in nuclear structure and dynamics. 

Future studies should determine whether accumulation of biological age-specific proteins such as nuclea lamina associated lamin proteins and Hutchinson–Gilford progeria syndrome (HGPS) inducing progerins could be a better predictor of survival and longevity as compared with chronological age. Since age-dependent cellular malfunctioning could also be determined by epigenetic modification that was highly dependent on the environmental conditions that the organism had experienced after birth, how epigenetic modification altered cell–nucleus interaction would be another challenge. We believe these studies could provide new pathological insight into cellular aging and the precise diagnosis of age-related chronic diseases.

## Supplementary Materials

The following are available online at https://www.mdpi.com/2072-666X/11/9/801/s1, Figure S1: Age dependent changes of cell morphology. Figure S2: Age dependent changes of cell motility. Figure S3: Age dependent changes of nucleus morphology.
