# Supplementary material for: Biological Aging Modulates Cell Migration via Lamin A/C-Dependent Nuclear Motion
Source: Micromachines (Basel). 2020 Aug 24;11(9):801. doi: 10.3390/mi11090801 (PMC7570206; doi:10.3390/mi11090801)
Supplement: Supplementary file 1 [file micromachines-11-00801-s001.pdf]

*micromachines*

*Supplementary Materials*

# Biological Aging Modulates Cell Migration via Lamin A/C-Dependent Nuclear Motion

**Jung-Won Park, Seong-Beom Han, Jungwon Hah, Geonhui Lee, Jeong-Ki Kim, Soo Hyun Kim and Dong-Hwee Kim**

**Supplemental Figure S1: Age dependent changes of cell morphology.**

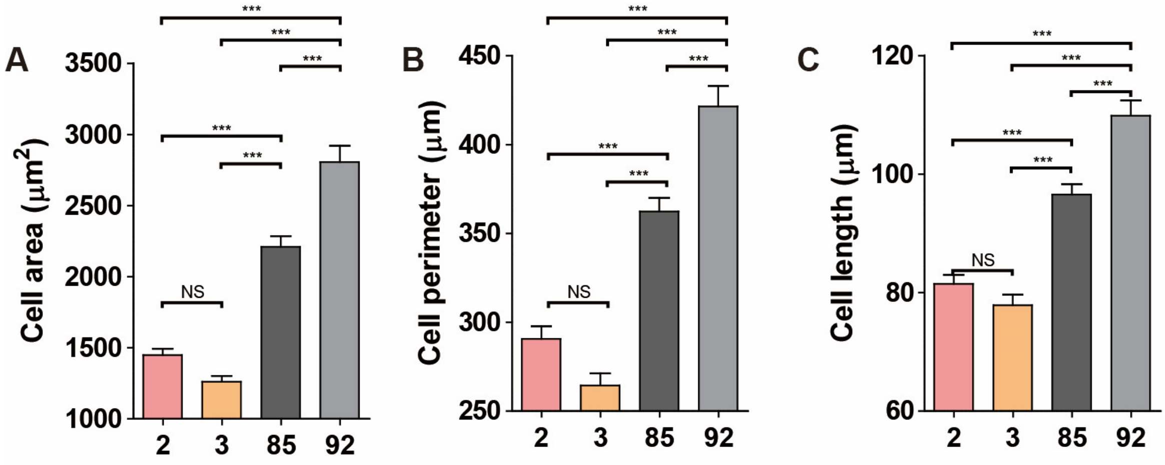


**Figure S1.** Old cells (age 85, 92) are larger than young cells (age 2, 3). These data are raw data for the Figure 1D, 1E, and 1F. X-axis depicts age of donors. > 290 cells were analyzed for each condition. (age 2: 310, age 3: 310, age 85: 311, age 92: 297) Error bars indicate SEM, and 1-way ANOVA using Tukey's test was applied (NS: not significant; ***: $p<0.001$).

**Supplemental Figure S2: Age dependent changes of cell motility.**

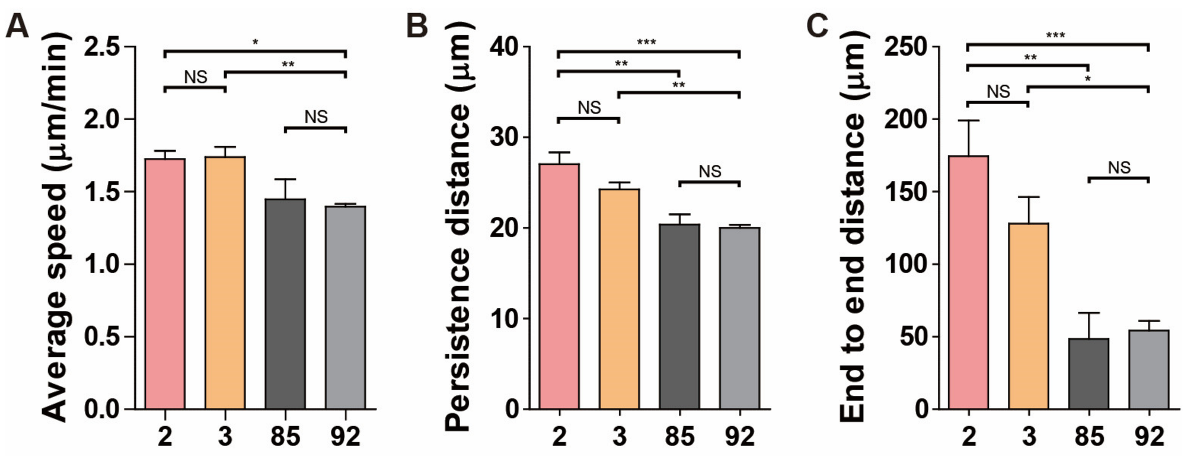


**Figure S2.** Old cells (age 85, 92) are less motile than young cells (age 2, 3). Cell dynamics are monitored for 8 h. These data are raw data for the Figure 1J–L. X-axis depicts age of donors. In each conditions, we analyzed more than 60 cells. (age 2: 16, age 3: 24, age 85: 6, age 92: 13) Error bars indicate SEM, and 1-way ANOVA using Tukey's test was applied (NS: not significant; *: $p<0.01$; **: $p<0.005$; ***: $p < 0.001$).

**Supplemental Figure S3: Age dependent changes of nucleus morphology.**

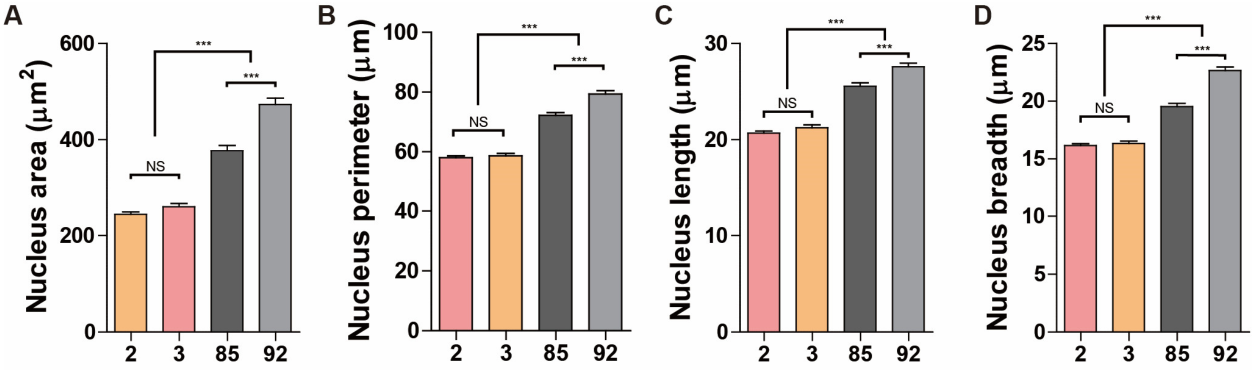


**Figure S3.** Nuclei of old cells (age 85, 92) are larger than young cells (age 2, 3). These data are raw data for the Figure 2D–G. X-axis depicts age of donors. > 290 cells were analyzed for each condition. (age 2: 310, age 3: 310, age 85: 311, age 92: 297) Error bars indicate SEM, and 1-way ANOVA using Tukey's test was applied (NS: not significant; ***: $p<0.001$).

© 2020 by the authors. Licensee MDPI, Basel, Switzerland. This article is an open access article distributed under the terms and conditions of the Creative Commons Attribution (CC BY) license (http://creativecommons.org/licenses/by/4.0/).
